# Enhancing Manufacturing Precision: Leveraging Motor Currents Data of Computer Numerical Control Machines for Geometrical Accuracy Prediction Through Machine Learning

**DOI:** 10.3390/s25010169

**Published:** 2024-12-31

**Authors:** Lucijano Berus, Jernej Hernavs, David Potocnik, Kristijan Sket, Mirko Ficko

**Affiliations:** 1 Intelligent Manufacturing Laboratory, Production Engineering Institute, Faculty of Mechanical Engineering, University of Maribor, Smetanova ulica 17, 2000 Maribor, Slovenia; jernej.hernavs@um.si (J.H.); d.potocnik@um.si (D.P.); kristijan.sket@um.si (K.S.); mirko.ficko@um.si (M.F.); 2 Lab3D Laboratory, Rudolfovo–Science and Technology Centre Novo Mesto, Podbreznik 15, 8000 Novo Mesto, Slovenia

**Keywords:** smart production machines, data-driven manufacturing, machine learning algorithms, CNC controller data, geometrical accuracy

## Abstract

Direct verification of the geometric accuracy of machined parts cannot be performed simultaneously with active machining operations, as it usually requires subsequent inspection with measuring devices such as coordinate measuring machines (CMMs) or optical 3D scanners. This sequential approach increases production time and costs. In this study, we propose a novel indirect measurement method that utilizes motor current data from the controller of a Computer Numerical Control (CNC) machine in combination with machine learning algorithms to predict the geometric accuracy of machined parts in real-time. Different machine learning algorithms, such as Random Forest (RF), k-nearest neighbors (k-NN), and Decision Trees (DT), were used for predictive modeling. Feature extraction was performed using Tsfresh and ROCKET, which allowed us to capture the patterns in the motor current data corresponding to the geometric features of the machined parts. Our predictive models were trained and validated on a dataset that included motor current readings and corresponding geometric measurements of a mounting rail later used in an engine block. The results showed that the proposed approach enabled the prediction of three geometric features of the mounting rail with an accuracy (MAPE) below 0.61% during the learning phase and 0.64% during the testing phase. These results suggest that our method could reduce the need for post-machining inspections and measurements, thereby reducing production time and costs while maintaining required quality standards.

## 1. Introduction

Digitalization is gaining traction among manufacturers. This shift is evidenced by the formulation of manufacturing strategies such as Industry 4.0 in Germany, the Industrial Internet initiative in the US, and China’s Made in China 2025 vision. These initiatives champion the adoption of modern information technologies (new-IT) within manufacturing, propelling the evolution toward smart manufacturing [1]. The objective of smart manufacturing is to transform the data gathered throughout the product lifecycle into actionable manufacturing insights, with the aim of generating favorable outcomes across all facets of manufacturing [2]. The central role of data is indicated by more than 1000 exabytes of annual growth of data generated by the modern manufacturing industry [3] and is based on the review by Tao et al. [4], which revolutionized the manufacturing sector, signaling a transformative shift towards an era of smart manufacturing embedding new-IT concepts such as Internet of Things (IoT) [5,6,7], cloud computing [8] and Artificial Intelligence (AI) [9,10,11]. This new-IT shift goes beyond pure technological development and marks a fundamental change in the operational, innovative, and value-adding paradigms of manufacturing companies. In this regard, AI is facilitating the rapid evolution of smart manufacturing through the integration of machine learning (ML) and deep learning (DL) with a data-driven approach [12].

Machining or subtractive manufacturing has been a cornerstone of manufacturing for an extensive period, still its relevance and adoption in today’s industry persists to grow [13]. Intelligent machining represents an industrial implementation of a data-driven smart manufacturing approach, leveraging the broad specter of data emitting from Computer Numerical Control (CNC) machine components (e.g., motor data). Contemporary research focuses on transforming this information into actionable insights. Smart machines, equipped with sensors and advanced analytical capabilities, are increasingly capable of real-time monitoring and predictive manufacturing. This innovation signifies a move from solely automated manufacturing towards more autonomous, efficient, and intelligent manufacturing processes [14]. State-of-the-art research mainly focuses on workpiece condition monitoring (roughness [15], surface integrity [16], and waviness [17,18,19]), tool condition monitoring [20,21], process-machine interaction [22,23], and process sustainability [24,25,26].

In manufacturing, smart machines equipped with AI capabilities revolutionize traditional processes by integrating advanced algorithms and data-driven decision-making. AI empowers these machines to exhibit intelligent behavior, enabling them to analyze vast amounts of data from sensors and historical records to make real-time adjustments and optimizations during the machining process. Machine learning (ML), a subset of AI, further enhances smart machines by enabling them to learn from experience and adapt their behavior accordingly. AI and ML have been incorporated into different aspects of machining processes, such as drilling, where AI has significantly enhanced efficiency, decision-making, and problem-solving within the oil and gas industry drilling operations [27]. Utilizing the neuro-fuzzy logic allowed Ranjan et al. [28] to develop a hole-quality prediction system. AI has been applied to automation, process control, real-time laser welding monitoring [29,30], and friction stir welding optimization with ANNs and fuzzy logic [31]. Moreover, it has been implemented in processes like turning [32], grinding [33,34], sawing [35], laser cutting [36,37], and additive manufacturing [38,39]. Given its usability and adaptability, AI has naturally been integrated into one of the most used machining technologies: milling. In this manufacturing process, ML algorithms can be utilized to predict tool wear, optimize cutting parameters, and enhance product quality by analyzing various factors such as material properties, cutting conditions, and tool characteristics. Ultimately, the synergy between smart machines, AI, and ML in milling manufacturing results in increased efficiency, precision, and productivity while reducing downtime and waste, paving the way for the next generation of manufacturing technologies. Various models and techniques have been developed to predict machining errors and workpiece deformation with high precision [40,41]. Machine learning-based models, such as Random Forest and Regression Tree, have been utilized to predict surface roughness and tool flank wear accurately [42]. Additionally, the combination of the metabolic grey model (MGM) with a nonlinear autoregressive (NAR) neural network has shown promising results in predicting machining errors with high accuracy, speed, and robustness [43]. Other commonly used ML algorithms in the literature include support vector machine (SVM) [44,45,46,47], Random Forest (RF) [48], backpropagation neural network (BpNN) [49,50,51,52], adaptive neuro-fuzzy inference system (ANFIS) [53], and probabilistic neural network (PNN) [54].

By applying machine learning algorithms to motor data, subtle changes in equipment conditions can be detected early, predicting wear, part accuracy, and potential failures before they occur. This proactive approach not only enhances reliability but also optimizes the overall manufacturing process, ensuring consistent quality and minimizing downtime. This paper tackles the prediction of part accuracy based on data gathered directly from the CNC controller. Different ML algorithms (KNN, DT, and RF) with ROCKET feature selection are compared based on RMSE, MAE, and MAPE.

The remainder of this paper is organized as follows. Section 2 outlines the proposed procedure, detailing the processed part and its overall structure, the machining process, and the control measurement device used. It also describes the collection and preprocessing of controller data and part accuracy measurements, followed by feature extraction techniques and the machine learning algorithms applied. Section 3 presents an analysis of the gathered controller data and its preprocessing as time-series data, the pairing of controller data with part accuracy measurements, and the outcomes of applying machine learning methods. Finally, Section 4 summarizes the findings and implications of the study, highlighting its contributions and potential for future applications.

## 2. Materials and Methods

### 2.1. Processed Part and Overall Structure of the Proposed Procedure

**Figure 1:**
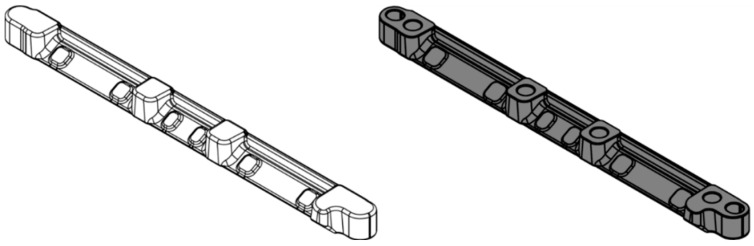
Process input, forged part (**left**) and process output, machined part (**right**).

**Figure 2:**
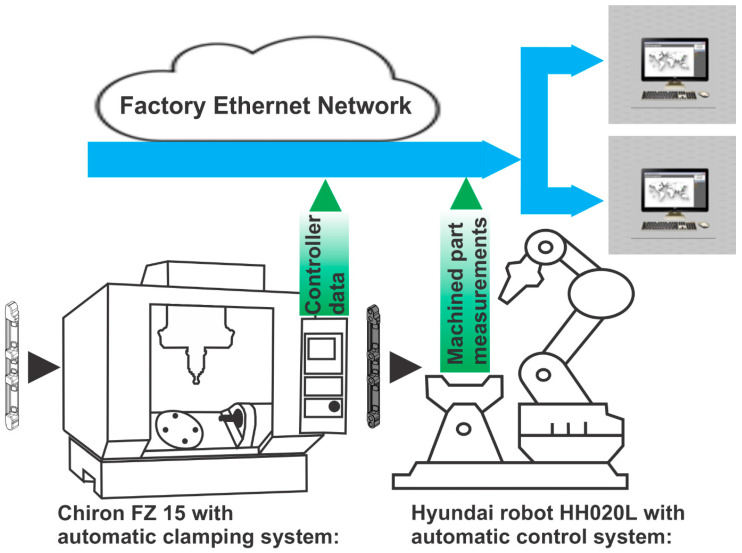
Block diagram of the proposed method.

The competitiveness of a machining process is dependent on two main factors: productivity and costs. Geometrical measurements and control of machined parts add unproductive time to the total process time and represent a bottleneck that lowers manufacturing cell productivity. This work will focus on a prediction of geometrical measurements of the mounting rail part (Figure 1), which is CNC-milled and geometrically inspected in a measurement cell (Figure 2).

The mounting rail is forged (not the subject of this study) before it is machined in the manufacturing cell. The part is made from a material (16MnCrS5 1.7139) that makes forging and machining challenging. It is used to attach the inlet manifold on the turbocharger side of the engine block. In addition to its fastening function, it is extremely important that it enables linear expansion due to high-temperature changes during the rail’s operation. The operating temperature rises from the initial normal room temperature to more than 400 °C in seconds (Table 1).

Mass production and the highest quality demands are typical traits of the automotive industry. The shape of forged parts becomes final after machining, and, therefore, a dimensional and geometrical check is necessary to exclude all parts that do not correspond to the customer’s geometrical tolerances. The manufacturing cell consists of the following:

– Two-spindle 4-axis milling machines, Chiron DZ15W, with pallet changer. Milling machines are general-purpose without any adaptations for the manufacturing cell. The machines are equipped with Fanuc 3li-model B5 controllers.
– An automatic clamping system. Clamping is performed by an automated hydraulic clamping device.
– Hyundai robot HH010L, which serves the manufacturing cell. This 6-axis industrial robot executes clamping/unclamping parts in the milling machine and placing them in/from the control station.
– An automatic control station for 100% control of the machined parts. This uses measurement probes from the Keyence company.

Machining is carried out on a 4-axis machine with two spindles that can machine two parts at once for maximal productivity. After processing, the parts are robotically removed from the machine and measured on an automated control station. The manufacturing cell is integrated into the Factory Ethernet Network, which is the most promising enabling technology for future industrial networks [56]. The process data from CNC machining and control cell measurements are monitored and stored in the cloud. Data from the Fanuc controller is captured via the Focas system. Process signals from each part’s machining are recorded as a time-dependent series (controller data) and data from the measurement device as multiple single-value variables (machined part measurements).

### 2.2. Machining Process and Control Measurement Device

**Figure 3:**
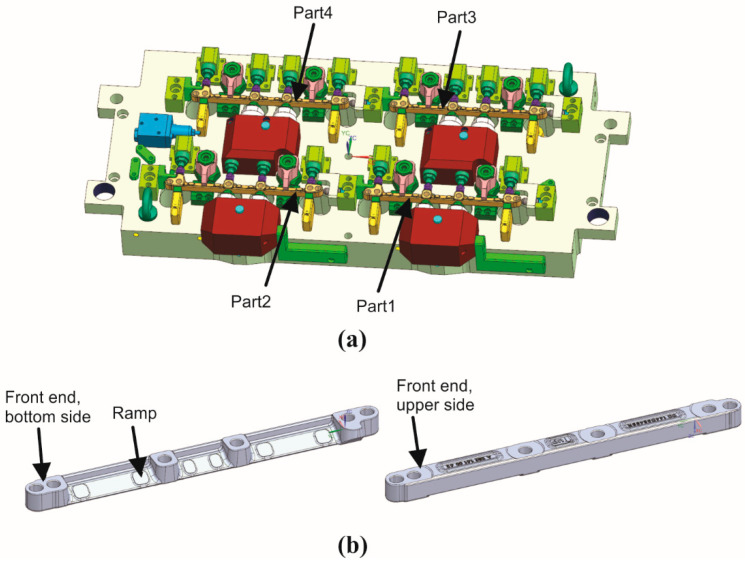
Automatic clamping device with 4 machined parts setting (**a**), machined part’s bottom and upper side features (**b**).

Machining is set to be performed in a realistic mass production environment wherein 4 machined parts are processed within a single clamping on an automatic clamping device (as shown in Figure 3a). Parts are milled with two spindles (HSK A63 holder) simultaneously in pairs, where Part 1–Part 2 represents the first, and Part 3–Part 4 represents a second pair. Machining consists of 8 operations, namely: 1. chamfer drilling, 2. drilling, 3. ramp milling, 4. bottom face milling, 5. ellipse milling, 6. chamfer milling, 7. top face milling, and 8. chamfer milling. During each operation, different tools with appropriate machining parameters are deployed, as shown in Table 2. The duration of the entire machining process is approximately 215 s to manufacture the part’s distinct features on the bottom and upper sides (as shown in Figure 3b). The clamping device needs to be rotated after the 6th operation and back again (to its start position) after finishing with the 8th operation.

**Figure 4:**
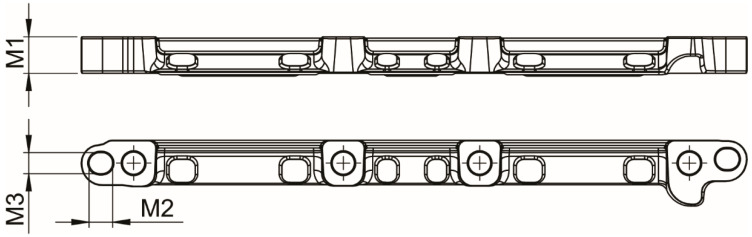
Relevant part measurements M1, M2 and M3.

Measurements of machined parts are performed with an automatic control station, which uses Keyence GT2 series high-accuracy digital contact displacement sensors (probes). The digital signals were acquired through KEYENCE (Belgium, Mechelen) NR-600 DAQ, and collected data is automatically saved and transferred to a server using the FTP client function. After milling all 4 parts, the Hyundai robot HH020L positions each machined part (Part 1, Part 2, Part 3, and Part 4) on the control station for M1, M2, and M3 measurements shown in Figure 4. Table 3 offers additional information on the sensors used and part measurements (M1, M2, and M3) relations.

### 2.3. Controller Data, Part Accuracy Measurements, and Data Preprocessing

Controller data are gathered from the Chiron DZ15W CNC controller Fanuc 31i-model B5 with an Ethernet-based protocol. In this paper, Focas Library with InfluxDB is used to enable real-time cloud-based data collection. Controller data, presented in Table 4, are recorded during machining for 13 different parameters and stored in the InfluxDB database. Each unique recording of these 13 different parameters represents the machining data for 4 machined parts (Part 1, Part 2, Part 3, and Part 4). Parameters in Table 4 can be grouped as a process (stored as date–time objects, integer, and string variables) and motor-related data (stored as time series).

**Figure 5:**
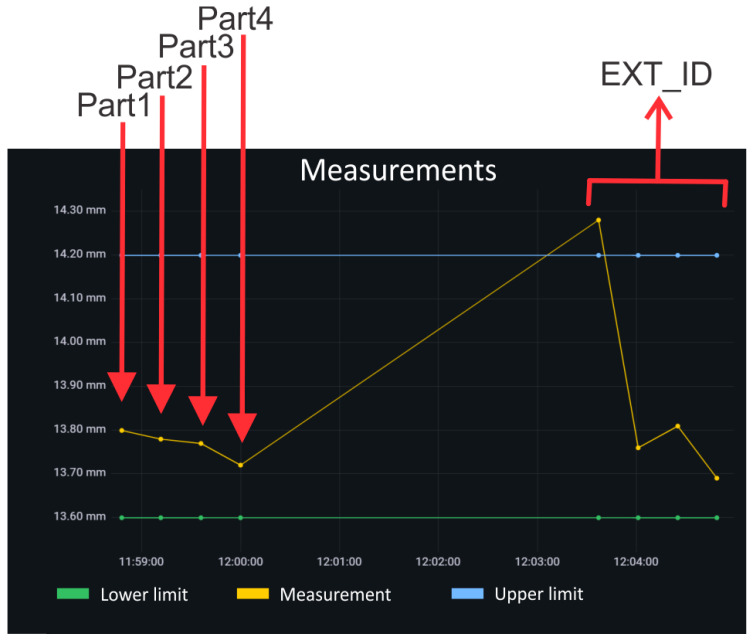
Grafana visualization for M1 measurements of Part 1, Part 2, Part 3, and Part 4 (with the same EXT_ID).

Part accuracy measurements are gathered from the automatic control station (after the robot picks the machined parts one after another, starting with Part 1 and ending with Part 4) and saved to the InfluxDB database. Part accuracy measurements, presented in Table 5, are recorded for 6 different parameters wherein each machined part (Part 1, Part 2, Part 3, and Part 4) gets assigned a unique combination of 3 process parameters (TIME, EXT_ID, and MEASUREMENT_ID) and 3 machined part measurements (M1, M2, and M3). Figure 5 shows M1 measurements in real-time with the Grafana visualization tool.

### 2.4. Feature Extraction

For tasks involving classification and regression, the relevance of extracted features is crucial. This is because an excess of unimportant features can impair the algorithm’s ability to generalize beyond the training data, which leads to overfitting. Overfitting occurs when a model learns the details and noise in the training data to such an extent that it negatively impacts its performance on new data. Therefore, it is important to ensure that the features used in the model are indicative of the underlying patterns and are not simply noise or irrelevant data. A well-curated set of relevant features helps to create models that are more accurate and robust [57]. For feature extraction from time series (controller data) data, we applied time series feature extraction based on scalable hypothesis tests (Tsfresh) and Random Convolutional Kernel Transformation (ROCKET).

Tsfresh is a Python package that combines 63 different methods for characterizing time series and computes a total of 794 time series features, which are then filtered using hypothesis tests tailored to the specific type of problem—either classification or regression—and the feature type, either categorical or continuous. This automation of feature selection ensures statistical significance and relevance, which is crucial to prevent the overfitting of ML models and achieve generalizability. Furthermore, Tsfresh is designed to efficiently process large amounts of time series data, allows for the customization and addition of new feature extraction algorithms, and can be integrated into parallel processing [58].

ROCKET is an approach for the feature extraction of time series that achieves a high degree of accuracy with considerably less computational effort than conventional methods. In this method, time series data is transformed using many random convolution kernels. These kernels vary in size, weighting, bias, dilation, and padding so that they can capture a wide range of features relevant to specific tasks. Among the most important aspects of ROCKET are its scalability and speed. The computational complexity of ROCKET is linear with the length of the time series and the number of training examples. One of the unique features of ROCKET is the use of the maximum value and the proportion of positive values from the feature maps resulting from convolution. This combination of features extracted from each kernel provides a comprehensive representation of the time series data for classification tasks. The resulting features can then be used to train classification or regression ML models [59].

### 2.5. Machine Learning Algorithms

For our computer modeling, we used five different ML algorithms to achieve the best possible correlation between our sensory data and the geometric accuracy of the parts, given that ML algorithms examine previous and current data to predict future outcomes. The methods used were

– Random Forest,
– K-nearest neighbors, and
– Decision Trees.

Random Forest (RF) combines ML algorithms with a set of tree classifiers, where each tree unit votes for the most popular class, and the results of this voting consequently provide a classification result. RF is characterized by robust properties, including high classification accuracy, good tolerance to outliers and noise, and avoidance of overfitting. It can be used for different types of ML problems (classification and regression) in various scientific fields such as medicine, bioinformatics, management science, economics, etc. It can handle both continuous and categorical variables, which further enhances its usability [60].

The k-nearest neighbors (k-NN) classification is a method where examples are classified based on the nearest neighbors’ classes, using a number, k, of such neighbors for decision-making. This approach, which utilizes the entire training dataset at runtime, often involves methods like majority voting or distance-weighted voting for class determination. The technique calculates the distance between a query and training samples, with weights applied to each feature, to select the k nearest neighbors. The class of the query is then determined either by majority or by weighing the votes of the neighbors based on their proximity. This method is flexible, supporting various distance metrics and can be adapted for both classification and regression tasks, where outputs are categorical or continuous, respectively. The effectiveness of k-NN can be enhanced by considering different similarity measures, computational optimizations, and dimension reduction techniques to improve performance and accuracy [61].

Decision Trees (DTs) are a versatile tool used in data mining for classification by recursively splitting the instance space into simpler, non-overlapping regions. The simplicity of DTs makes them understandable to non-experts as they visually represent decisions and their outcomes in a tree-like structure. Each internal node of a tree tests an attribute and divides the data into further subsets, while each leaf node returns a classification decision, often in the form of a class label or a probability vector. These trees can handle both numeric and nominal attributes and are, therefore, suitable for a wide range of data types. DTs do not require assumptions about the distribution of the data and can deal effectively with errors and missing values. However, they are subject to limitations, such as a tendency to overfit and a drop in performance for datasets with complex interactions between attributes. Nevertheless, DTs remain a widely used classification technique [62].

## 3. Results

### 3.1. Analysis of Gathered Controller Data and Time-Series Preprocessing

**Figure 6:**
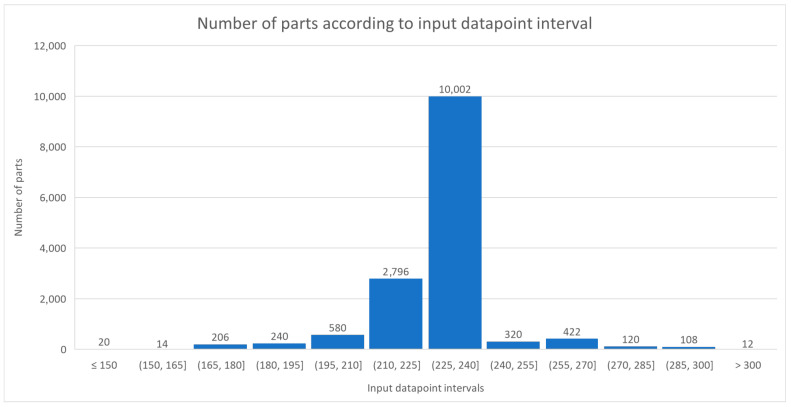
Number of parts according to input datapoint intervals.

The total number of machined parts for which data was collected from the machine controller amounted to 14,840. Upon reviewing the data for the processed parts, it was found that they do not have the same number of datapoints, which is crucial for further processing with machine learning algorithms. Significant discrepancies in the number of captured datapoints between individual processed parts were observed, as illustrated in the histogram (Figure 6). Most processed parts (10,002) had a datapoint count in the range of 225 to 240 datapoints.

A more detailed analysis of the data based on descriptive statistics is presented in Table 6. It was found that there are machined parts with a minimum of 68 datapoints and a maximum of 322 datapoints. The average number of datapoints (229) was not present in any of the parts. To conclude the data analysis, it is worth noting that the largest proportion of parts (11.01%) had 231 datapoints.

**Figure 7:**
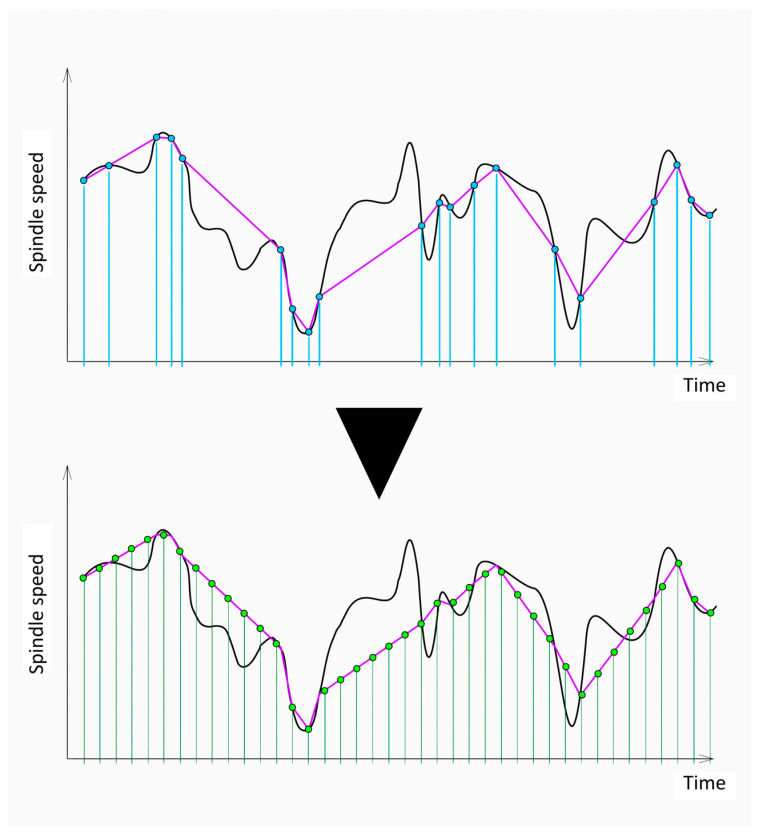
Preprocessing of controller data time series. The linear interpolation between the acquired datapoints was executed first and is depicted on the top, followed by resampling of the data with an evaluation of exactly 231 chronologically equidistant points. This operation ensures the same number of datapoints in each sample.

Controller data is comprised of various numbers of datapoints due to a non-fixed sampling rate. Such a dataset with different-sized samples is unsuitable for building machine learning algorithms, as each sample is expected to have an exact and predefined form. The rectification of this problem starts with the analysis of acquired data, with the aim of determining what number of datapoints should be all samples should conform to. The analysis of gathered data shows that the modus is 231 datapoints in a sample. First, linear interpolation was performed between existing datapoints. The total time of each sample was discretized to 230 regular intervals, simulating the fixed sampling rate. Next, the new signal values were generated by evaluating the interpolation function at every inter-equidistant time increment. This operation yielded uniform samples, each of which possessed exactly 231 datapoints. The steps of preprocessing are collectively presented in Figure 7.

### 3.2. Pairing of Controller Data and Part Accuracy Measurements

Pairing controller data with part accuracy measurements is an essential step to provide inputs and outputs, which will be used for machine learning. The input–output pair connects the motor parameters (presented in Table 4) and machined part measurements (presented in Table 5) based on process parameters (presented in Table 4 and Table 5). Each input–output pairing gets assigned a unique identification name composed of EXT_ID, TIME, and MEASUREMENT_ID parameters. Wherein EXT_ID is associated with a certain batch of parts (in association with the clamping device), TIME is used for traceability, and MEASUREMENT_ID is used to enable the assignment of data to specific machined part in the batch (exact part on the clamping device).

Inputs–outputs pairing is created only if all the requirements specified in this paragraph are met. Firstly, specific EXT_ID needs to be present in controller data (inputs) and in part accuracy measurements (outputs). Secondly, all the subprogram numbers representing all eight machining operations (presented in Table 2) need to be recorded in the database (under the SUB_PROGRAM parameter in Table 4). Thirdly, each subprogram needs to be repeated for both Part1–Part2 and Part3–Part4 pairs (indicated by CODE_LINE_NUMBER parameter drop inside specific SUB_PROGRAM) since machining of each pair is achieved with the same subprogram but different work coordinate systems–WCS (for example WCS G54 for Part 1–Part 2 and G57 for Part 3–Part 4).

### 3.3. Machine Learning

Supervised machine learning enables the input–output relationship modeling. In this study, input variables represent the motor parameters (nine time-series data gathered from the Fanuc controller), while outputs represent the machined part measurements (three continuous variables gathered from the automatic control station). To extract the features needed for the regression ML algorithms, feature extraction is performed on controller data (motor parameters) with Tsfresh and ROCKET algorithms. Extracted features serve as inputs for various ML algorithms. A total of 6110 datapoints were used for the analysis with a K-fold cross-validation scheme. Regression ML algorithms such as Random Forest (RF), Support Vector Regression (SVR), k-nearest neighbor (k-NN), and Decision Trees (DT) were used to model the outputs (machined parts measurements). Feature extraction and ML are performed with the use of Python’s Tsfresh and scikit-learn libraries. Table 7 shows the detailed settings (hyper-parameters) of used ML algorithms with fixed and grid-searched parameters. Fixed parameters are the hyper-parameters, which are kept fixed during the ML modeling, wherein grid-searched parameters are evaluated in all the possible combinations of parameter values, and the best combination is retained combinatorically. The RF procedure is grid searched with 30 different combinations (five instances of the “maximum tree depth” hyper-parameter and six instances of the “number of estimators”). SVR’s, k-NN’s, and DT’s modeling based on grid search is performed on 35, 100, and 20 combinations of different hyper-parameter settings.

**Figure 8:**
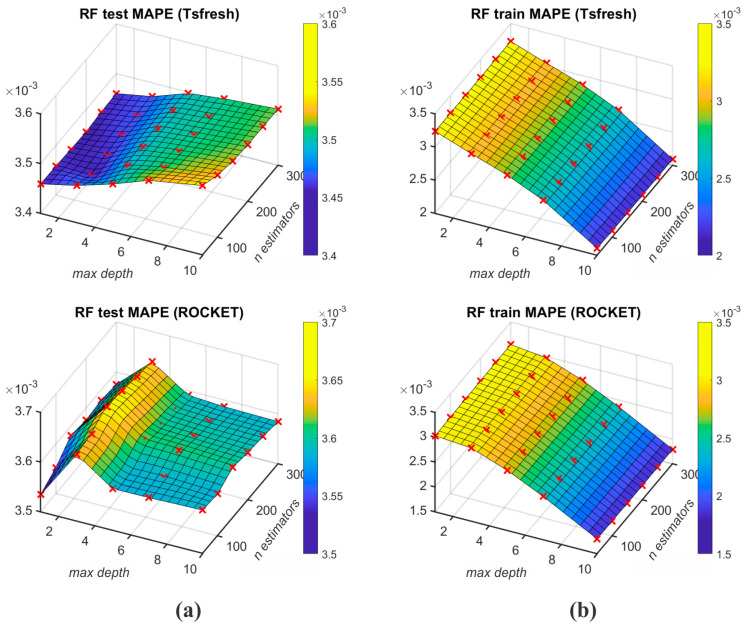
Test MAPE (**a**) and train MAPE (**b**) of RF’s grid searched parameters for n_estimators and max_depth.

Table 8 displays the K-fold cross-validation results of ML algorithms (RF, SVR, k-NN, and DT) for different feature extraction protocols, namely Tsfresh and ROCKET. To enable robust generalisation to the unseen data, the K-fold cross-validation is used (K=10) to evaluate the performance of ML algorithms with mean test and mean train MAPE metrics. The best mean test MAPE results for M1 output were achieved with the use of the RF algorithm, wherein Tsfresh and ROCKET feature extractions achieved 0.003455 and 0.003535. Figure 8 depicts the RF algorithm MAPE results for different grid searched parameters. The worst mean test MAPE is achieved by the k-NN algorithm for both Tsfresh and ROCKET feature extraction protocols (respectively, 0.003731 and 0.003755). The best mean train MAPE was achieved with the DT algorithm, wherein the Tsfresh and ROCKET feature extractions achieved 0.002823 and 0.002783.

**Figure 9:**
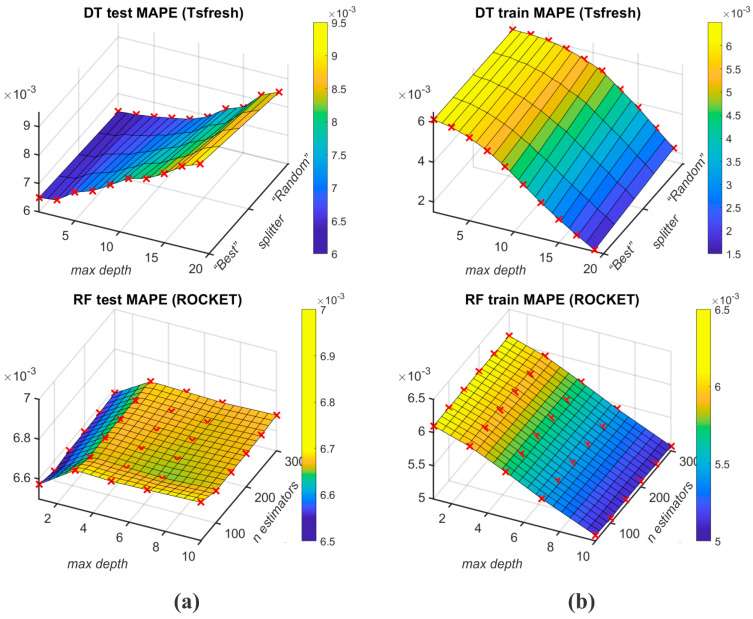
Test MAPE (**a**) and train MAPE (**b**) of DT’s and RF’s grid searched parameters for splitter, n_estimators, and max_depth.

Table 9 displays K-fold cross-validation (K=10) results of ML algorithms (RF, SVR, k-NN, and DT) for Tsfresh and ROCKET feature extraction protocols. The best mean test MAPE results for M2 output were achieved with the use of DT and RF algorithms, wherein DT with the Tsfresh feature extractions achieved 0.006369, and RF with the ROCKET feature extraction achieved 0.006550. Figure 9 depicts the DT-Tsfresh and RF-ROCKET results of test and train MAPE for different grid searched parameters. The worst mean test MAPE (0.006765) with the Tsfresh feature extraction algorithm is achieved by k-NN, while DT achieved the worst results (0.006930) using the ROCKET feature extraction. The best mean train MAPE was achieved with the k-NN algorithm, wherein the Tsfresh and ROCKET feature extractions achieved 0.006049 and 0.005999.

**Figure 10:**
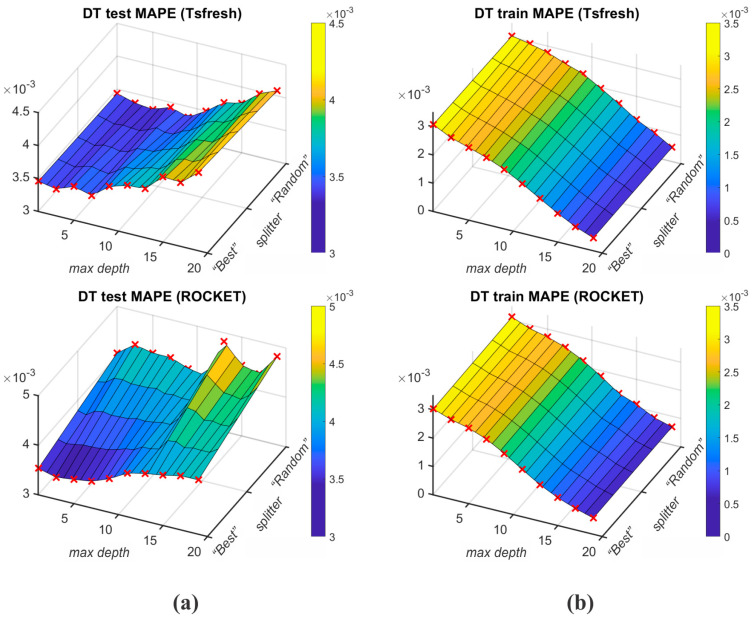
Test MAPE (**a**) and train MAPE (**b**) of DT’s grid searched parameters for splitter and max_depth.

Table 10 displays K-fold cross-validation results of ML algorithms (RF, k-NN, and DT) for the Tsfresh and ROCKET feature extraction protocols. The best mean test MAPE results for M3 output were achieved with the use of the DT algorithm, wherein the Tsfresh-based feature extraction resulted in 0.003325, and ROCKET achieved a 0.003443 mean test MAPE. Figure 10 depicts the DT algorithm MAPE results for different grid searched parameters. The worst mean test MAPE was achieved by the k-NN algorithm for both Tsfresh and ROCKET feature extraction protocols (respectively, 0.003571 and 0.003457). The best mean train MAPE was achieved with the RF algorithm, wherein Tsfresh and ROCKET feature extraction resulted in 0.002753 and 0.002629.

Similarly, as mentioned before, the K-fold cross-validation of DT, k-NN, and RF with Tsfresh and ROCKET-based feature extraction also the Support Vector Regression (SVR) and Stochastic Gradient Descent (SGD) algorithms with Tsfresh and ROCKET-based feature extraction were performed. The use of SVR and SGD-based K-fold cross-validation (K = 10) resulted in negative MAPE values (for M1, M2, and M3), indicating either the inappropriateness of presented interpolation-based preprocessing or the inability of certain ML algorithms (namely SVR and SGD) for predictive modeling of the presented problem. Additional evaluation metrics, such as mean squared error (MSE) and maximum error (ME) of DT (for predictive modeling of M2 and M3) and RF (for M1) with Tsfresh-based feature extraction, are depicted in Table 11.

## 4. Discussion

Direct verification of machine parts’ geometric accuracy with 3D scanners and coordinate measuring machines represents a challenge for the mass production industry. Aspects of this challenge usually include employing highly educated human resources and costly machines on repetitive tasks, resulting in increased production time and costs.

This study presents a novel approach to gathering the data directly from a CNC controller during multiple machined parts clamping and measuring the part’s accuracy measurements on an automatic control station. Since the size of the time-series controller data varies while the feature extraction and ML algorithms need fixed inputs, the interpolation-based preprocessing of data is adopted to provide equally sized inputs. Since the real production environment experiment is conducted, the gathered either input–output pair needs to be complete and existing. This requirement is ensured with the presented pairing protocol, which enables every input, has all machining operations (subprograms) present, and that the appropriate part measurements (among four parts on clamping device) are assigned to the right controller data time-series. These preprocessed input–output pairs are in combination with different feature extractions (Tsfresh and ROCKET) and different ML (DT, k-NN, and RF) algorithms used for regression-based predictive modeling. The regression models are evaluated based on K-fold cross-validation with 10 folds (K = 10) and MAPE metric with different settings of hyper-parameters. DT and RF algorithms with Tsfresh feature extraction achieved the lowest test MAPE. ROCKET-based feature extraction with selected ML algorithms was able to successfully model the input–output relationship but achieved higher MAPE values in comparison with Tsfresh feature extraction.

In the future, additional research should be directed into the application of other feature extraction methods, additional ML algorithms, and different preprocessing (interpolation) techniques.

## Figures and Tables

**Table 1:** Chemical composition of steel 16MnCrS5 (in weight %) [55].

Carbon	Silicon	Manganese	Phosphorus	Sulfur	Chrome	Iron
0.14–0.19	max 0.4	1–1.3	max 0.025	0.02–0.04	0.8–1.1	Balance

**Table 2:** Machining process and tool information.

Operation Number	Operation Description (Part Side, Clamping Rotation)	Tool Description (Tool Number)	Spindle Speed- (mm/min)	Feed Rate (mm/min)	Operation Time (s)	Part 1–2 or Part 3–4 Pairing	Subprogram Number
1	Chamfer drilling (bottom, A0)	Chamfer drill ϕ8.54 (T10)	2335	557	23	Part 1–2	O4113
						Part 3–4	O4113
2	Drilling (bottom, A0)	Drill ϕ7.6 (T8)	2775	610	11	Part 3–4	O4104
						Part 1–2	O4104
3	Ramp milling (bottom, A28)	End mill ϕ12 (T12)	2387	764	24	Part 1–2	O4102
						Part 3–4	O4102
4	Bottom face milling (bottom, A0)	End mill ϕ12 (T12)	2387	764	58	Part 3–4	O4101
						Part 1–2	O4101
5	Ellipse milling (bottom, A0)	End mill ϕ6 (T11)	3289	395	37	Part 1–2	O4105
						Part 3–4	O4105
6	Chamfer milling (bottom, A0)	Minimaster ϕ10 (T9)	6000	299	09	Part 3–4	O4116
						Part 1–2	O4116
7	Top face milling (upper, A180)	End mill ϕ12 (T12)	2387	764	53	Part 3–4	O4107
						Part 1–2	O4107
8	Chamfer milling (upper, A180)	Minimaster ϕ10 (T9)	6000	299	21	Part 1–2	O4108
						Part 3–4	O4108

**Table 3:** Contact displacement sensors and machined part measurements.

Machined Part Measurement	Additional Info	Sensor Type
M1—part’s thickness	The probe is directed downwards, with a low probability of residue accumulation.	Keyence GT2-A32
M2—hole length	The probe is installed horizontally; its tip is protected by the cover.	Keyence GT2-PA12
M3—hole width	The probe is installed horizontally; its tip is occluded by the casing of a measuring rod.	Keyence GT2-PA12

**Table 4:** Controller data.

	Parameter	Description	Unit
**Process** **parameters**	TIME	Absolute time	/
	EXT_ID	Fixture table identification number	/
	SUB_PROGRAM	Current CNC subprogram	/
	CODE_LINE_NUMBER	Current line number in the subprogram	/
**Motor** **parameters**	FEED_RATE	Feed rate	mm/min
	SPINDLE_SPEED_S1	Rotational velocity of spindle S1	min^−1^
	SPINDLE_SPEED_S2	Rotational velocity of spindle S2	min^−1^
	SPINDLE_LOAD_S1	Torque on the spindle S1, expressed in el. current	A
	SPINDLE_LOAD_S2	Torque on the spindle S2, expressed in el. current	A
	SERVO_LOAD_CURRENT_X	el. current in the motor for X axis	A
	SERVO_LOAD_CURRENT_Y	el. current in the motor for Y axis	A
	SERVO_LOAD_CURRENT_Z	el. current in the motor for Z axis	A
	SERVO_LOAD_CURRENT_A	el. current in the motor for A axis	A

**Table 5:** Part accuracy measurements.

	Parameter	Description	Unit
**Process** **parameters**	TIME	Absolute time	/
	EXT_ID	Fixture table identification number	/
	MEASUREMENT_ID	Identification number of parts on the fixture table in sequence	/
**Machined part measurements**	M1	Part thickness	mm
	M2	Hole length	mm
	M3	Hole width	mm

**Table 6:** Results of descriptive statistics.

Function	Nr. of Datapoints	Nr. of Parts	Ratio (Nr. of Parts/All Parts)
**min**	68	2	0.01%
**max**	322	2	0.01%
**average**	229	0	0.00%
**median**	230	1418	9.56%
**modus**	231	1634	11.01%

**Table 7:** Detailed settings of ML algorithms.

ML Algorithm	Fixed Hyper-Parameters	Grid Searched Hyper-Parameters
RF	ccp_alpha: 0.0, criterion: squared error, max_features: 1.0, max_leaf_nodes: None, max_samples: None, min_impurity_decrease: 0.0, min_samples_leaf: 1, min_samples_split: 2, min_weight_fraction_leaf: 0.0, oob_score: False, random_state: 54, warm_start: False	max⁡_depth: [1, 3, 5, 7, 10] and n_estimators: [50, 100, 150, 200, 250, 300]
k-NN	Algorithm: auto, metric: minkowski, metric_params: None, p: 2	leaf_size: [10, 20, 30, 40, 50] , n_neighbors: [1, 3, 5, 7, 9, 11, 13, 15, 17, 19] and weights: [uniform, distance]
DT	ccp_alpha: 0.0, criterion: squared error, max_features: None, max_leaf_nodes: None, min_impurity_decrease: 0.0, min_samples_leaf: 1, min_samples_split: 2, min_weight_fraction_leaf: 0.0, random_state: 54, warm_start: False	max_depth:[1, 3, 5, 7, 9, 11, 13, 15, 17, 19] and splitter: [best, random]

**Table 8:** MAPE of different ML algorithms and feature extraction protocols for M1.

	Feature Extraction Algorithm	ML Algorithm	Best Grid Searched Hyper-Parameters	Mean Test MAPE	Mean Train MAPE
M1—machined part measurement	Tsfresh	**RF**	max_depth=1 n_estimators=200	**0.003455**	**0.003234**
		k-NN	leaf_size=10 n_neighbors=19 weights=uniform	0.003731	0.003349
		DT	max_depth=7 splitter=random	0.003474	0.002823
	ROCKET	**RF**	max_depth=1 n_estimators=50	**0.003535**	**0.003029**
		k-NN	leaf_size=10 n_neighbors=19 weights=uniform	0.003755	0.003276
		DT	max_depth=5 splitter=best	0.003595	0.002783

**Table 9:** MAPE of different ML algorithms and feature extraction protocols for M2.

	Feature Extraction Algorithm	ML Algorithm	Best Grid Searched Hyper-Parameters	Mean Test MAPE	Mean Train MAPE
M2—machined part measurement	Tsfresh	RF	max_depth=1 n_estimators=50	0.006378	0.006109
		k-NN	leaf_size=10 n_neighbors=19 weights=uniform	0.006765	0.006049
		**DT**	max_depth=1 splitter=random	**0.006369**	**0.006144**
	ROCKET	**RF**	max_depth=1 n_estimators=100	**0.006550**	**0.006102**
		k-NN	leaf_size=10 n_neighbors=19 weights=uniform	0.006760	0.005999
		DT	max_depth=1 splitter=random	0.006930	0.006163

**Table 10:** MAPE of different ML algorithms and feature extraction protocols for M3.

	Feature Extraction Algorithm	ML Algorithm	Best Grid-Searched Hyper-Parameters	Mean Test MAPE	Mean Train MAPE
M3—machined part measurement	Tsfresh	RF	max_depth=3 n_estimators=300	0.003337	0.002753
		k-NN	leaf_size=10 n_neighbors=17 weights=uniform	0.003571	0.002914
		**DT**	max_depth=5 splitter=random	**0.003325**	**0.002775**
	ROCKET	RF	max_depth=5 n_estimators=150	0.003626	0.002629
		k-NN	leaf_size=10 n_neighbors=19 weights=uniform	0.003457	0.002837
		**DT**	max_depth=3 splitter=best	**0.003443**	**0.002814**

**Table 11:** Evaluation metrics of best-performed ML algorithms for M1, M2, and M3 machined part measurements.

Machined Part Measurements	ML Algorithm	Best Grid-Searched Hyper-Parameters	Mean Test MSE	Mean Train MSE	Mean Test ME	Mean Train ME
M1	RF	max_depth=1 n_estimators=200	0.013185	0.004093	1.070290	1.422961
M2	DT	max_depth=1 splitter=random	0.022739	0.008752	1.125031	2.664294
M3	DT	max_depth=5 splitter=random	0.022985	0.007967	1.333249	0.114721

## Data Availability

Data are contained within the article.
